# Construct strength of a drilling trajectory angled proximally avoiding the posterior interosseous nerve in single incision distal biceps repair; a comparative cadaveric study

**DOI:** 10.1016/j.xrrt.2026.100665

**Published:** 2026-01-22

**Authors:** Arno A. Macken, Igor Shirinskiy, Nynke van der Gaast, Wouter J. van der Poel, Ronald L.A.W. Bleys, Derek F.P. van Deurzen, Pieter Caekebeke, Denise Eygendaal, Michel P.J. van den Bekerom

**Affiliations:** a Alps Surgery Institute, Clinique Générale d’Annecy, Annecy, France; b Department of Orthopaedics and Sports Medicine, Erasmus MC, Rotterdam, Netherlands; c Afdeling Chirurgie, Radboud University Medical Center, Nijmegen, Netherlands; d Department of Clinical Anatomy, Faculty of Medicine, Utrecht University, Utrecht, Netherlands; e Department of Orthopaedics, OLVG Amsterdam, Amsterdam, Netherlands; f Orthopaedisch Centrum Limburg, Genk, Belgium; g Department of Movement Sciences, Vrije Universiteit Amsterdam, Amsterdam, Netherlands

**Keywords:** Distal biceps tendon, Biceps reinsertion, Biceps repair, Radius, Bicortical button, Posterior interosseous nerve, Cadaveric study, Biomechanics

## Abstract

**Background:**

Distal biceps tendon repair using a bicortical button is a biomechanically robust technique but carries a risk of posterior interosseous nerve (PIN) injury, particularly with traditional drilling trajectories. Cadaveric studies have suggested that angling the drill trajectory both ulnarly and proximally may increase the distance to the PIN and reduce this risk. However, the biomechanical construct strength of such modified trajectories remains unestablished.

**Methods:**

Sixteen fresh-frozen human upper limbs were randomly assigned to either a control group (20° ulnar and perpendicular) or an intervention group (20° ulnar and 30° proximal). Standardized bicortical button repairs were performed following simulated distal biceps ruptures. All specimens underwent cyclic loading (1,000 cycles, 5-100 N, 2.5 Hz), followed by load-to-failure testing. Primary outcome was maximum load to failure. Secondary outcomes included construct stiffness, displacement, and mode of failure.

**Results:**

Two failures occurred during cyclic loading, both in the control group (*P* = .164). There were no significant differences in load to failure (control: median 247.2 N [range: 210.1-310.9]; intervention: 284.0 N [149.5-308.5]; *P* = .345), stiffness (control: 56.2 N/mm; intervention: 53.3 N/mm; *P* = .852), or displacement during cyclic loading or at failure (*P* > .3). Failure modes included suture rupture, tendon–suture interface failure, and bone fracture; all were classified as type 1 failures. No statistically significant differences in failure mode were observed between groups, although bone-related failure occurred only in the intervention group.

**Conclusion:**

A proximally angled (30°) and ulnar (20°) drilling trajectory for distal biceps repair using a bicortical button yields construct strength comparable to the traditional perpendicular axial ulnar trajectory. This technique may reduce the risk of PIN injury without compromising mechanical integrity. Further clinical studies are warranted to confirm safety and efficacy in vivo.

Several techniques have been described for repairing a distal biceps tendon rupture, usually involving unicortical or bicortical drilling. Bicortical button technique resulted in the highest load to failure based on a cadaveric study.^10^ Two recent meta-analyses of biomechanical studies confirmed that a bicortical button results in the strongest construct.^4^^,^^17^ Adding an interference screw to the construct does not increase construct strength but is associated with a higher rate of tendon failure.^4^^,^^17^

An important drawback of the bicortical drilling technique is the risk of posterior interosseous nerve (PIN) damage when creating the drill hole or by entrapping the nerve behind the button.^2^^,^^8^ In a previous study, a PIN injury occurred in 22% (6/27) cases, of which 4 were transient.^1^ Iatrogenic PIN palsy causes severe disability due to loss of motor function of the extensors of the fingers, while in most cases, extension of the wrist remains intact.^19^

Traditionally, a hole is drilled from the bicipital tuberosity perpendicularly through the midline of the radial shaft. However, previous cadaveric studies have identified drilling trajectories that minimize the risk of PIN damage by changing the drilling angle. A drilling angle deviating 20° toward the ulnar side has been shown to increase the distance to the PIN (Fig. 1), which is commonly advised for current practice.^2^^,^^7^^,^^18^ Similarly, a drilling angle up to 30° proximally is identified as a safer trajectory, resulting in a greater distance from the PIN (Fig. 2).^2^^,^^3^^,^^11^^,^^14^ To our knowledge, this proximally angled drilling trajectory is currently less frequently studied or adopted in practice.

**Figure 1:**
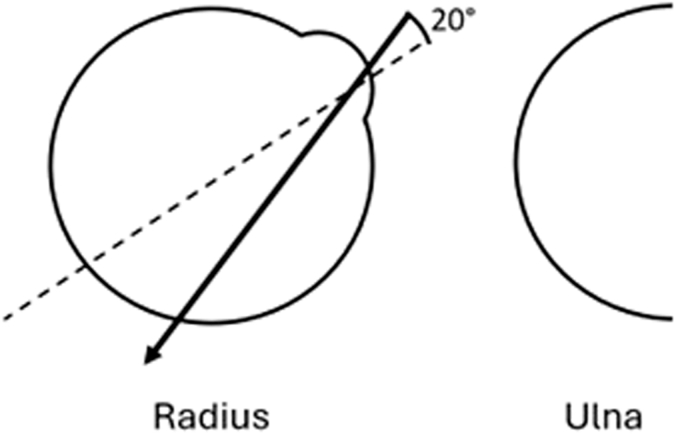
Axial view of the radius showing the drilling trajectory starting from the center of the ulnar half of the bicipital tuberosity, aiming 20° to the ulnar side.

**Figure 2:**
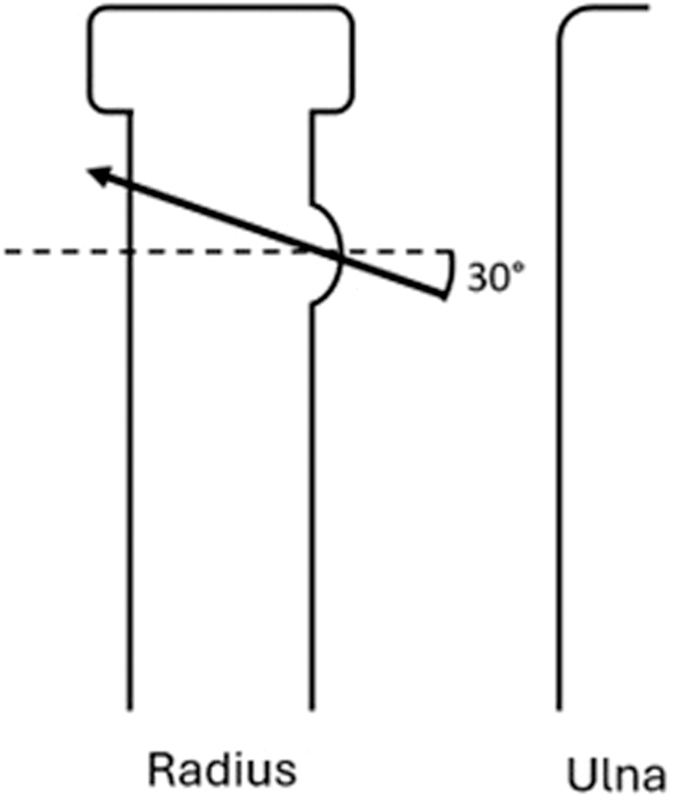
Lateral view of the radius showing the drilling trajectory starting from the bicipital tuberosity, aiming 30° to the proximal side.

To our knowledge, there are no studies comparing the construct strength between the traditional perpendicular ulnar drilling trajectory (perpendicular to the radius and 20° ulnar) and the trajectory avoiding the PIN (30° proximal and 20° ulnar; Figs. 1 and 2). Therefore, this study aimed to compare the maximum load to failure of the construct between the traditional perpendicular ulnar drilling trajectory and the novel trajectory avoiding the PIN in distal biceps repair using a bicortical button in human cadavers. The secondary aims were to assess and compare the mode of failure, displacement, and construct stiffness between the 2 groups.

## Methods

### Subjects

This study was performed using fresh-frozen human upper limbs. The specimens were obtained from cadavers collected by the Department of Anatomy of University Medical Center Utrecht through a donation program. Before the donors were deceased, written informed consent was obtained detailing the use of their entire bodies for educational and research purposes. For this study, only limbs were included that did not reveal any obvious signs of previous injury and could achieve full elbow flexion and extension as well as full pronation and supination. In total, 16 limbs were available for this study and randomly assigned between the 2 treatment groups (control group: standard perpendicular drilling and intervention group: PIN avoiding proximal drilling). Simple block randomization was performed, stratifying by side (left or right limb) to ensure an equal proportion in each group.

### Preparation and technique

For all specimens, the elbow was placed in 30° of flexion and 90° of forearm supination. The proximal radius was approached from the volar side, leaving the interosseous membrane of the forearm, elbow joint capsule, and ligaments intact. A complete distal biceps rupture was simulated by making a standardized transverse cut with a scalpel to detach all tendon fibers. For both groups, a 3.2-millimeter hole was drilled through both cortices, starting from the center of the ulnar half of the bicipital tuberosity, aiming 20° toward the ulnar side in the axial plane (Fig. 1). For the control group, the drilling trajectory was aimed perpendicular to the cortex in the longitudinal plane. For the intervention group, the drilling trajectory was aimed 30° proximally in the longitudinal plane. (Fig. 2). A 4.5-millimeter drill was then used to widen the volar hole in the first cortex.

**Figure 3:**
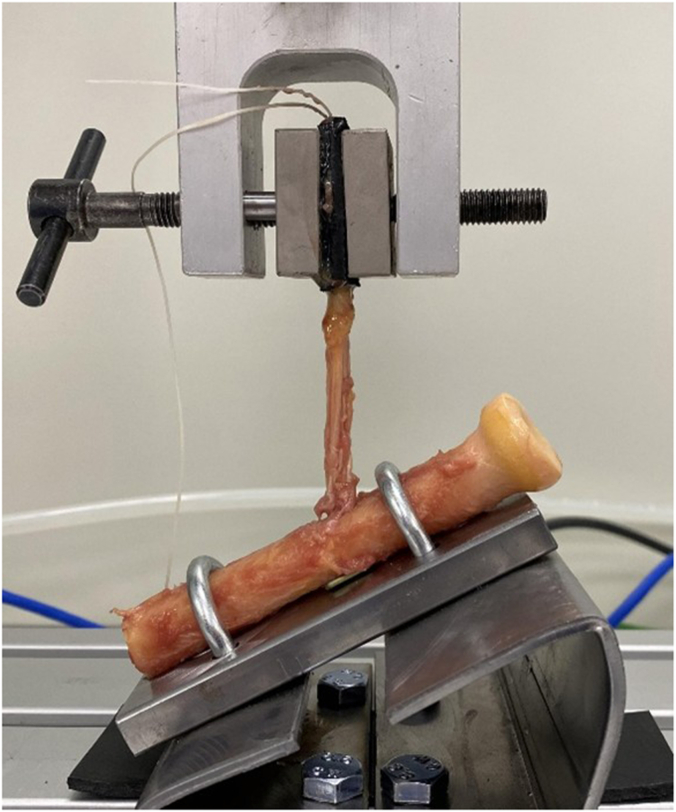
Picture of the set-up used for testing repaired distal biceps specimens.

The tendon stump was prepared using a nonabsorbable suture (ULTRABRAID◊ (#2); Smith and Nephew, Watford, England, UK), passed through the tendon in a Krackow stitch fashion with 4 rows of continuous locking sutures, and then passed through the button (ENDOBUTTON; Smith and Nephew, Watford, England, UK). Next, the button was passed through both cortices, and the sutures were pulled to flip and secure the cortical button against the dorsal cortex of the radius. The sutures were tightened to pull the stump into the volar hole and secured firmly. After performing the button placement, all soft tissues were removed except for the distal biceps tendon and the radius was sawed off at least 4 centimeters distal to the biceps reinsertion site, leaving a proximal radius of at least 8 centimeters and a distal biceps tendon of at least 8 centimeters (Fig. 3). After soft-tissue removal, correct position of the button against the cortex was confirmed.

### Testing and outcomes

The proximal radius was clamped inside a pneumatic modular frame (LiTeM, Ancona, Italy) with a vertical double-column testing machine and TC-Micro series load cell (L-TC-M-500) to a custom mount and the distal biceps tendon was firmly attached to a metal clamp 2 null centimeters distal to the fixation site of the biceps tendon (Fig. 3). The mount was angled at 30°, simulating the physiological loading condition with the elbow in slight extension. All specimens were subjected to a cyclic loading test consisting of 1,000 cycles at 2.5 Hertz (Hz), ranging from 5 to 100 Newton (N). After the 1,000 cycles were completed, the load was returned to 5 N. For displacement measurements, the position of the actuator was recorded before and after cyclic testing. The specimens that did not fail during the cyclic loading protocol were subjected to a continuously increasing load until failure at an extension rate of 10 Newton per second (N/s). The maximum load to failure was recorded at the peak of loading power before failure of the construct. The mode of failure was determined by observing and, if necessary, by dissecting the failed construct. If needed, multiple modes of failure were recorded for one case. The type of failure was classified as type 1 (caused by inadequate construct integrity, such as failure at tendon–implant interface) and type 2 (caused by native tissue damage with or without construct failure, typically proximal to the zone of fixation).^17^

### Statistical analysis

Data were extracted and analyzed using Statistical Package for the Social Sciences (SPSS Inc., Chicago, IL, USA). The normality of the data distribution was assessed by visual inspection of QQ plots and the Kolmogorov–Smirnov test. Depending on the distribution, an independent samples *t*-test or a Mann–Whitney *U* test was performed to compare the ultimate load to failure, displacement, and stiffness. Stiffness of the construct was calculated using the linear portion of the load–displacement graph from the load-to-failure testing. The mode of failure was compared between the groups using Fisher exact tests. A *P* value lower than .05 was considered significant.

## Results

### Cohort

All 16 specimens were included in the data analysis. The control and intervention groups both consisted of 4 right elbows (50%) and 4 left elbows (50%).

### Outcome

In total, 2 constructs (14.3%) failed during cyclic loading, with both failures occurring in the control group and none in the intervention group (*P* = .164). The remaining 14 constructs (87.5%) all completed the load-to-failure test. There were no significant differences in the displacement after cyclic loading, displacement at failure, ultimate load to failure, or construct stiffness (*P* > .345; Table I). The modes of failure were not significantly different between the 2 groups (*P* > .077, Table I). All failures were classified type 1: failure at the suture–tendon interface, suture breakage, or a fracture of the bone at the site of the button. All failures were related to the biceps tendon repair; there were no cases of unrelated failures, such as a radius fracture or technical failure.

**Table I:** Outcomes.

Variable	Control (n = 8)	Intervention (n = 8)	*P* value
Cyclic testing			
Displacement after cyclic loading, mm, median (range)	24.4 (12.3-40.6)	16.2 (11.0-22.6)	.414
Load-to-failure testing			
Displacement at failure, mm, median (range)	36.5 (20.0-68.3)	29.9 (23.5-54.7)	.366
Ultimate load, N, median (range)	247.2 (210.1-310.9)	284.0 (149.5-308.5)	.345
Stiffness, N/mm, median (range)	56.2 (30.9-60.9)	53.3 (31.7-80.3)	.852
Mode of failure, n (%)			
Suture	4	6	.608
Tendon	4	0	.077
Bone	0	2	.467

*mm*, millimeters; *N*, Newton.

## Discussion

This cadaveric study aimed to assess the construct strength of distal biceps repair using a bicortical button, comparing a traditional perpendicular ulnar drilling trajectory with a trajectory aimed ulnar and proximal to avoid the PIN. Mechanical testing replicating the repetitive stress of elbow movement through cyclic loading was used to assess the construct resistance to regular motion and post-operative activity. Load-to-failure testing was used to measure mechanical strength in demanding circumstances. The results from both cyclic loading and load-to-failure tests suggest that the performance of the proximally angled drilling trajectory is similar to the traditional perpendicular ulnar trajectory, suggesting that the proximal ulnar trajectory might be a useful technique to potentially reduce the risk of damaging the PIN. However, the adjusted angle may be technically more difficult than standard perpendicular drilling.

The clinical relevance of the drilling trajectory remains disputed. Several cadaveric studies have shown that a drilling trajectory aimed perpendicular to the cortex and 30° ulnar leads to a larger distance from the guidewire to the PIN.^2^^,^^3^^,^^11^^,^^14^ Similarly, some studies reported that drilling in a proximally aimed angle without ulnar deviation also results in a larger distance between the guidewire and the PIN.^2^^,^^7^^,^^11^^,^^18^ However, few studies have addressed the combined ulnar and proximal angle.^2^ Furthermore, clinical studies that assess the rate of postoperative PIN palsy are scarce, and a comparative analysis would require a large cohort due to the rare occurrence of PIN palsy. Based on the current literature, the proximal ulnar angle seems to be the safest trajectory; however, this remains to be proven in more thorough studies. Besides avoiding the PIN, the proximal angle increases the tunnel length, whereas the ulnar angle decreases the tunnel length. The tunnel length was not measured in this study. In our experience, there is no practically relevant difference in tunnel length.

To our knowledge, this is the only study comparing the strength of different drilling trajectories. Previous studies assessing load to failure of the bicortical button repair using a perpendicular trajectory report similar results to the current study, with maximum load to failure ranging between 191.1 and 439.6 N, compared to 247.2 and 284.0 N in the current study.^5^^,^^6^^,^^9^^,^^10^^,^^12^^,^^13^^,^^15^^,^^16^ The considerable variation in mean load to failures reported in previous literature is likely due to the variation in repair techniques, testing constructs, and testing protocols. This complicates comparing the current results with the literature. However, it can be concluded that the load to failure using the ulnar and proximally angled drilling trajectory is not inferior to the literature.

Notably, a torn suture was noted as the failure mode in both groups. However, a torn tendon at the tendon–suture interface as the mode of failure only occurred in the control group, whereas a break or fissure in the cortical bone leading to failure of the construct only occurred in the intervention group. These differences were not statistically significant, but the failure mode may be clinically important. Previous biomechanical studies did show that using nonlocking stitches and more sutures reduced the chance of tendon failure, potentially due to sutures cutting through the tendon.^17^ Previous cadaveric studies assessing the construct strength of the perpendicular drilling trajectory mention all 3 modes of failures (suture, tendon, bone), with suture failure being the most common in most studies.^5^^,^^6^^,^^9^^,^^13^^,^^15^ The density and shape of the cortex behind which the button is placed may differ between the 2 locations. Larger studies are required to assess the failure mode between 2 drilling trajectories.

### Limitations

The results of this study should be interpreted alongside its inherent limitations. Firstly, this was a time-zero, in-vitro biomechanical study; therefore, natural healing of the tendon and bone is not taken into account. In addition, the construct strength of the straight perpendicular drilling trajectory was not included in the comparison. However, due to the broad adoption of the ulnar angled trajectory, which is considered safer, the straight perpendicular angle was considered outside the scope of this study. Moreover, the distance to the PIN was not measured in this study. Instead, the safety of the drill trajectories was based on previous cadaveric studies. Furthermore, no information was available regarding the human cadaveric specimens used for this study. These specimens were donated for medical research, and details on the donors were blinded to the researchers. However, based on the average life expectancy in the Netherlands (70-90 years), it can be assumed that the age of the specimens used in this study is likely comparable to those used in similar previous studies. The results of this study cannot be directly extrapolated to a different population. In addition, our study included a relatively small sample size. This inherent challenge of working with cadaveric specimens is consistent with previous biomechanical studies. Moreover, the mode of failure was determined by inspecting the specimen after testing, which is subject to the researchers' interpretation. Furthermore, construct strength was tested in a static position rather than a dynamic model of elbow motion, which may limit the accuracy and external applicability. Last, our displacement measurements were derived from the actuator position rather than from image-based tracking of the tendon and bone. This means we were unable to determine the precise source of displacement within the construct.

## Conclusion

In this cadaveric study on distal biceps repair using a bicortical button, the drilling trajectory aimed 20° ulnar and 30° proximally showed similar strength compared to the traditional perpendicular ulnar trajectory, suggesting that this technique may aid in reducing the risk of PIN damage while maintaining construct strength. However, clinical studies are required to confirm these results.

## Disclaimers:

Funding: No external funding was received for this study.

Conflicts of interest: D.F.P. van Deurzen has received institutional support from Smith and Nephew and has given paid presentations for Stryker. M.P.J. van den Bekerom has received institutional support from Smith and Nephew. The other authors, their immediate families, and any research foundation with which they are affiliated have not received any financial payments or other benefits from any commercial entity related to the subject of this article.
